# Topical Administration Effect of *Sargassum duplicatum* and *Garcinia mangostana* Extracts Combination on Open Wound Healing Process in Diabetic Mice

**DOI:** 10.1155/2022/9700794

**Published:** 2022-02-09

**Authors:** Dwi Winarni, Fitria Nikmatul Husna, Muhammad Farraz Syadzha, Raden Joko Kuncoroningrat Susilo, Suhailah Hayaza, Arif Nur Muhammad Ansori, Mochammad Amin Alamsjah, Muhamad Nur Ghoyatul Amin, Pugar Arga Christina Wulandari, Pratiwi Pudjiastuti, Khalijah Awang

**Affiliations:** ^1^Department of Biology, Faculty of Science and Technology, Universitas Airlangga, Surabaya 60115, Indonesia; ^2^Doctoral Program in Veterinary Science, Faculty of Veterinary Medicine, Universitas Airlangga, Surabaya 60115, Indonesia; ^3^Department of Marine, Faculty of Fisheries and Marine, Universitas Airlangga, Surabaya 60115, Indonesia; ^4^Department of Chemistry, Faculty of Science and Technology, Universitas Airlangga, Surabaya 60115, Indonesia; ^5^Department of Chemistry, Faculty of Science, University of Malaya, Kuala Lumpur 50603, Malaysia

## Abstract

This research aimed to determine the topical administration effect of the combination of *Sargassum duplicatum* and *Garcinia mangostana* extracts to ameliorate diabetic open wound healing. The study used 24 adult males of *Mus musculus* (BALB/c strain, 3–4 months, 30–40 g). They were divided into normal control groups (KN) and diabetic groups. The diabetic group was streptozotocin-induced and divided further into three treatment groups: the diabetic control group (KD), the *S. duplicatum* treatment group (PA), and the combination of *S. duplicatum* and *G. mangostana* treatment group (PAM). The dose of treatment was 50 mg/kg of body weight. Each group was divided into three treatment durations, which were 3 days, 7 days, and 14 days. The wound healing process was determined by wound width, the number of neutrophils, macrophages, fibroblasts, fibrocytes, and collagen density. Histological observation showed that the topical administration of combination extracts increased the re-epithelialization of the wounded area, fibroblasts, fibrocytes, and collagen synthesis. The topical administration of combination extracts also decreased the number of neutrophils and macrophages. This study concluded that the topical administration of the combination of *S. duplicatum* and *G. mangostana* extracts improved the open wound healing process in diabetic mice.

## 1. Introduction

Diabetes mellitus (DM) is a group of metabolic disorders characterized by hyperglycemia caused by abnormalities in the process of insulin secretion, insulin performance, or both [1]. There was a prevalence of about 9.6% of the world's diabetes population in 2013, and it tends to increase until 9.9% in 2035 [2]. Hyperglycemia is associated with increased reactive oxygen species (ROS), which triggers an increase in oxidative stress [3]. Hyperglycemia leads to the accumulation of ROS in tissues or cells with different metabolic pathways, such as activation of the polyol pathway, formation of advanced glycation end products (AGEs) and activation of its receptor, activation of isoform protein kinase C (PKC), increased activity of hexosamine pathway, and decreased antioxidant defenses. The accumulation of ROS can cause an oxidant-endogenous antioxidant imbalance which triggers the activation of sensitive intracellular signaling pathways stress, and this condition triggers cell damage and causes diabetes complications [4].

The wound healing process involves the following phases: the inflammatory phase (on day 3), the proliferation phase (on day 7), and the remodeling phase (on day 14) [5]. Various cells that play roles in wound healing include neutrophils, monocytes, macrophages, fibroblasts, B cells, T cells, mast cells, and endothelial cells [6]. Those cells are actively involved in producing and regulating various cytokines and growth factors [7]. Monocytes, which later turn into macrophages, are the primary producers of proinflammatory cytokines, such as IL-1*β* (interleukin-1*β*), TNF-*α* (tumor necrosis factor-*α*), and IL-6 (interleukin-6). Monocytes also produce growth factors, such as VEGF (vascular endothelial growth factor), IGF-1 (insulin-like growth factor-1), and TGF-*β* (transforming growth factor-*β*) in both diabetes and nondiabetes conditions, which are the essential contributors in wound healing. Neutrophils, keratinocytes, fibroblasts, mast cells, and endothelial cells are also significant producers of cytokines such as TNF-*α* and IL-10 and growth factors such as VEGF, IGF-1, and TGF-*β* [6]. Diabetes leads to an impaired wound healing process, which due to the condition of hyperglycemia can increase ROS and trigger oxidative stress, thus interfering with each phase, which negatively affects the long-term quality of life, such as increased morbidity and mortality [7]. Hyperglycemia and oxidative stress change the epigenic code resulting in changes in the polarization and modulation of macrophages.

The inability to polarize macrophages is one of the main reasons the wound healing process is delayed [8]. Excessive production of proinflammatory cytokines can cause impaired angiogenic responses and microvascular complications, interfere with macrophage function and neutrophils, interfere with migration and proliferation of keratinocytes and fibroblasts, and interfere with the process of factor production growth [9, 10]. High levels of proinflammatory cytokines in diabetes, such as IL-6 and TNF-*α*, cause disruption of the inflammatory, hyperinflammatory cascade, and insulin resistance [7]. Decreasing growth factors reduces proliferation and migration of fibrocytes and fibroblast cells. If the number of fibrocytes and fibroblasts goes down, then collagen synthesis also decreases. In addition, hyperglycemia can also inhibit the expression of endothelial nitric oxide synthase (eNOS) and increase nuclear expression factor-kappa B (NF-*κ*B) [6]. Decreased eNOS expression inhibits the migration of keratinocytes, thereby inhibiting the process of re-epithelialization that started at the proliferation phase. In the absence of re-epithelialization, a wound cannot be considered healed. Decreased eNOS expression also decreases VEGF so that angiogenesis is disrupted. These conditions can trigger tissue damage causing gangrene [11]. Given the lack of information about inflammatory cells in the wound healing process by topical administration, this study will only focus on the role of inflammatory cells in open wound tissue treated by a combination of natural products.

*S. duplicatum*, a hydrocolloid with strong absorption, absorbs a lot of rapid exudates in a gel form that creates a moist environment ideal to stimulate wound healing [10]. Alginate contains high calcium so that it can help in the process of blood clotting [11]. Moreover, alginate from *S. duplicatum* extracts has a polysaccharide component with a molecular weight of 3.535 × 10^4^ and antioxidant activity after being assayed by the DPPH test [12]. Closing the wound using alginate can absorb excess wound exudate, maintain a humid environment physiologically, and minimize bacterial infection in the wound area [13]. *G. mangostana* is potentially an antioxidant that can fight free radicals played by *α*-mangostin, which in the study by Wulandari et al., *G. mangostana* extract exhibited powerful antioxidant activity with an IC_50_ of 29.06 [14–17]. Therefore, this study aimed to determine the influence of a combination of *S. duplicatum* and *G. mangostana* on the wound width, the number of neutrophils, the number of macrophages, the number of fibroblasts, the number of fibrocytes, and the density of collagen in streptozotocin-induced diabetic mice.

## 2. Materials and Methods

### 2.1. Plant Identification

The material used in this study is the seaweed of *Sargassum duplicatum* and *Garcinia mangostana* pericarp, which were identified in the Plant Taxonomy Laboratory, Department of Biology, Universitas Airlangga, Surabaya, Indonesia.

### 2.2. *S. duplicatum* Extraction

The extraction procedures were referred to the Research and Development Center for Marine and Fisheries Product Processing and Biotechnology, Jakarta, Indonesia. Fresh *S. duplicatum* (50 g) was rinsed with water and added with 1.125 mL of 0.1% KOH [18]. Then, 1% HCL was added at a ratio of 1 : 30 w/v for 1 hour and filtered. The precipitated gel was then added with 2% Na_2_CO_3_ and 4% NaOCl. A 2-propanol solution was used to blanch the extract. After that, the extract was dried under the sun for 12 hours and then smoothened and weighed.

### 2.3*. G. mangostana* Extraction

We used the pericarp parts of the *G. mangostana* fruit. The pericarp was rinsed with water, blended, and macerated with ethanol. Then, the solvent was evaporated with a rotary vacuum evaporator at 50°C. The crude extract was collected, lyophilized, and weighed [19].

### 2.4. Diabetic Mice Induction

This study used healthy adult male mice (*Mus musculus*), strain BALB/c, aged 3–4 months, with a body weight ranging from 30–40 g. Mice were obtained from the Faculty of Pharmacy, Universitas Airlangga, Surabaya, Indonesia, and were acclimatized for two weeks in the Animal Laboratory, Faculty of Science and Technology, Universitas Airlangga, Surabaya, Indonesia. All animal procedures were carried out in compliance with the protocol approved by the Animal Care and Use Committee, Faculty of Veterinary Medicine, Universitas Airlangga (2.KE.049.04.2019). Fasting blood glucose levels were measured before and after lard and streptozotocin administration. All mice were in control of environmental conditions (25 ± 5°C, humidity of 50 ± 10%, and 12 light/dark cycles). Mice were provided with water and standard pellets *ad libitum*. Diabetes was induced in mice by multiple low-dose streptozotocin (STZ) with 30 mg/kg in citrate buffer pH 4 via injection intraperitoneally for five consecutive days following a high-fat diet for three weeks. Only mice with fasting blood glucose levels of more than 130 mg/dL were used as diabetic mice.

### 2.5. Animal Treatments

The research used a completely randomized design according to the previous studies [12, 14]. Mice were divided into normal control (KN) and diabetic groups. The diabetic group was further divided into three treatment groups: diabetic control (KD), *S. duplicatum* treatment group (PA), and a combination of *S. duplicatum* and *G. mangostana* treatment groups (PAM). Each group consisted of six mice. The treatment dose was 50 mg/kg of body weight. All mice were wounded by making a 1 cm section on the gluteal (under ethyl chloride local anesthesia), and this activity was considered day 0. Each group has three treatment durations of 3 days, 7 days, and 14 days. At the end of the treatment, those mice were sacrificed, anesthetized by ketamine (15 mg/kg body weight) and xylazine (2 mg/kg body weight) intramuscularly. Their skin was cut and proceeded for further histological observation.

### 2.6. Histological Observation

The histological observation was conducted on the cross section of wounded skin (5 mm thickness, hematoxylin-eosin (H and E) staining). The wound healing process was determined by wound width, the number of neutrophils (polymorphonuclear cells, lobulated nucleus, and small granules), macrophages (large cell, round cell, round nucleus, or vacuolated cytoplasm to support phagocytic activity), fibroblasts (large flat, irregular shape cell, oval shape of nucleus, or basophilic cytoplasm), fibrocytes (elongated nuclei, spindle shape, or acidophilic cytoplasm), and collagen density at the wound-healing site. Wound width was measured under the light microscope at 40× magnification, while others at 400× magnification were observed at the connective tissue in the wound healing site. ImageJ software was used to measure wound width and collagen density.

### 2.7. Statistical Analysis

All data were presented as mean ± SD and analyzed by one-way ANOVA followed by the Duncan test to evaluate significance between groups (*α* *=* 0.05) with SPSS 21 software (SPSS Ltd., Surrey, UK). There was a significant difference while *p* < 0.05.

## 3. Results

### 3.1. Effect of *S. duplicatum* and *G. mangostana* on Wound Healing Parameters

As shown in Table 1, on day 3 measurement, the diabetic control group (KD) showed a significant decrease (*p* < 0.05) in wound width, fibrocytes number, and collagen density in comparison with those of the normal control group (KN), but the group also showed a significant increase in neutrophils number and macrophages number. Inducing with *S. duplicatum* extract only (PA) exhibited significantly increased fibrocyte number and collagen density compared to those of the diabetic control group. Moreover, the *S. duplicatum* group also displayed a significant decrease in neutrophil numbers and macrophage numbers. In addition, when compared to the control group, the combination extracts group could significantly increase the fibrocytes number and collagen density in wound width while showing a significant decrease in neutrophils number and macrophages number. On day 7, the diabetic control group significantly increased in the wound width's neutrophil number and macrophage number parameters and significantly decreased in fibroblast number, fibrocyte number, and collagen density compared to those of the normal control group. Otherwise, the *S. duplicatum* extract group showed a significant decrease in the neutrophils number and macrophages number in wound width compared to the diabetic control group. However, the fibrocytes numbers and collagen density parameters exhibited contrary results, which significantly increased the effect of the extract. On day 14, the diabetic control group displayed a significantly increased neutrophil number and a significantly decreased fibrocyte number only after being compared with the normal control group. Therefore, no significant report was present in between *S. duplicatum* group and the diabetic control group, whereas the combination extracts group (PAM) showed a significant increase (*p* < 0.05) in fibroblast number and fibrocyte number in comparison with those of the diabetic control group.

### 3.2. Effect of *S. duplicatum* and *G. mangostana* on Histology of the Wound Healing Site

**Figure 1:**
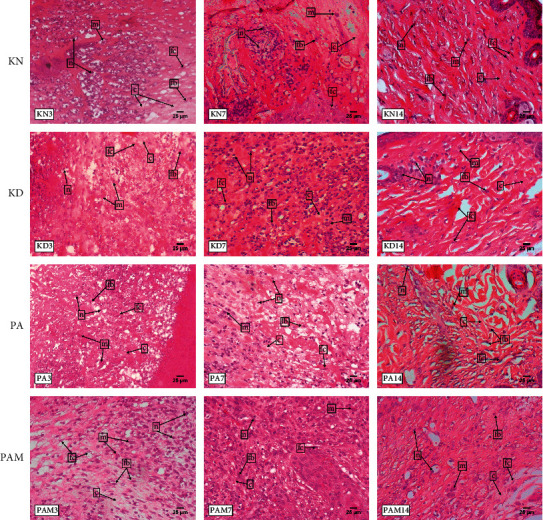
Wound healing parameters on day 3, 7, and 14 of treatment. Wound width (a), the number of neutrophils (b), the number of macrophages (c), the number of fibroblasts (d), the number of fibrocytes (e), and collagen density (f) in the open wound healing process in diabetic mice. Different letters show a significant difference based on the Duncan test (*α* < 0.05). KN: normal control, KD: diabetic control, PA: *S. duplicatum* extract treatment, and PAM: combination of *S. duplicatum* and *G. mangostana* extract treatment.

As shown in Figure 1, this histological analysis displayed re-epithelization during the wound healing process. On day 3, the KN group with no diabetic wound showed a fairly wide wound within the *epidermis*. Meanwhile, in the KD group has a wound diameter slightly wider than the KN group. The PA group displayed a tighter size diameter than the KD group, which is almost similar to the KN group. After that, the PAM group presented better treatment in reducing the diameter of the wound compared to other groups. On day 7, the wound diameter in the KD group was still large, whereas in another group showed improvement with a tighter size diameter. However, all groups showed equally well-wound closure on day 14.

### 3.3. Effect of *S. duplicatum* and *G. mangostana* on Histology of Connective Tissue

**Figure 2:**
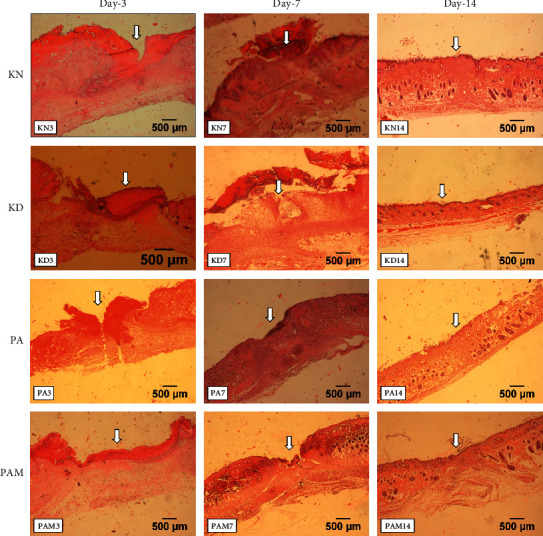
Histology of the wound healing site (pointed by white arrows). KN: normal control, KD: diabetic control, PA: *S. duplicatum* extract treatment, and PAM: the combination of *S. duplicatum* and *G. mangostana* extract treatment.

These results were related to connective tissue attribute numbers such as neutrophil, macrophage, fibroblast, fibrocyte, and collagen density, as shown in Figure 2. On day 3, the KD group presented the most increasing neutrophil and macrophage cell numbers when the healing process was still at the first stage, but fibroblast, fibrocyte, and collagen density were still less than other groups. On day 7, all groups had decreasing neutrophil and macrophage cells, with a still high number in the KD group. The fibroblasts, fibrocytes, and density collagen displayed an increase in numbers than before, whereas the PAM group gave the best growth. On day 14, all groups already produced collagen in the almost totally wounded area and massively decreased neutrophil, macrophage, fibroblast, and fibrocyte numbers in that wound area.

## 4. Discussion

Diabetic wounds are often experienced by many diabetic patients and are called the silent killers due to the difficulty of handling these wounds. Inflammatory cells are linked with wound healing mechanisms in the skin, such as neutrophils and macrophages. Both are associated with phagocytosis. After neutrophils have reached the end of their lifetime in tissue, they would be cleansed mostly by macrophages through the phagocytosis process [20]. In this study, neutrophils play an essential role in wound healing, producing lysozyme enzymes, and cleaning bacteria and various cell debris [5]. Neutrophils also play an essential role in producing cytokines such as TNF-*α* and IL-10 and growth factors such as VEGF, IGF-1, and TGF-*β* [6]. At 48 hours after the injury, the monocytes from the blood vessels around the wound move to the wound area and differentiate into macrophages activated through chemokine signaling. Monocytes are differentiating into macrophages and can help neutrophils in phagocytosis [21]. The monocyte, which turns into macrophages, is a significant producer of proinflammatory cytokines that stimulate the migration and proliferation of fibroblasts, endothelial cells, and keratinocytes to repair skin tissue damage [6, 13]. In the proliferation phase, fibroblasts are the primary cells in the tissue reconstruction process due to their ability to produce a certain amount of collagen needed in wound healing. Usually, fibroblasts are not active cells with metabolic activity and a proliferation rate, which tends to be slow. After the injury process, fibroblasts will become active cells proliferating rapidly and migrating to enter the injury area.

This result exhibited a proliferation process that becomes fibroplasia and produces collagen for reconstruction tissue [5]. Moreover, collagen synthesis is effective starting from day 7 after the injury. After that, the re-epithelialization process, which covers most of the keratinocytes, begins to migrate and experience stratification and differentiation to restructure the epidermal barrier function. The re-epithelialization process also increases extracellular matrix production, growth factors, cytokines, and angiogenesis through the release of growth factors such as keratinocyte growth factor [22]. The wound is healed after being supported by re-epithelialization. Diabetic conditions produce a severe infection, attracting more neutrophils and macrophages to clean debris cells and fight bacteria in the wound area. This mechanism is strengthened by infection due to high levels of glucose. In addition, neutrophils and macrophages will phagocyte bacteria, pathogens, and debris on the wound area, raising the number of both cells relatively higher than normal. The presence of high ROS levels caused a permanent hypoxic condition in the wound area. In addition, hyperglycemia and oxidative stress due to diabetes cause epigenic code mutations, resulting in polarization and macrophage modulation. The inability of macrophage polarization is one of the main reasons the wound healing process becomes postponed [8]. Diabetic conditions also inhibit the proliferation phase of the open wound healing process.

Excessive production of proinflammatory cytokines due to increasing ROS can inhibit the migration and proliferation of keratinocytes and fibroblasts and inhibit the synthesis of growth factors so that wound healing will be disrupted. Proinflammatory cytokine production due to increasing ROS causes angiogenic response disorders, microvascular complications, and disruption of macrophage and neutrophil functions [9, 10]. Moreover, these cytokines are involved in the migration and proliferation of keratinocytes, fibroblasts, and growth factor production. High levels of proinflammatory cytokines such as IL-6 and TNF-*α* in diabetes cause disruption of the inflammatory cascade, hyper inflammation, and insulin resistance [7]. Decreasing growth factors will decrease the proliferation and migration of fibrocytes and fibroblast cells. Hyperglycemia in people with diabetes also triggers active PKC pathways, which can inhibit the expression of eNOS and increase the expression of NF-*κ*B [4].

The decreased expression of eNOS inhibits the migration of keratinocytes, thereby inhibiting the re-epithelization process [11]. It suggests that the re-epithelization in people with diabetes was impaired due to increased ROS caused by hyperglycemia. Besides the increased ROS, the disruption of the re-epithelization process is also caused by infection from bacteria. The process indicates that in diabetics, ROS will cause the growth factor-producing cells to malfunction. *S. duplicatum* can absorb excess wound exudate, maintain a physiologically humid environment, and minimize bacterial infections in the wound area. However, it does not have a maximal effect because the number of neutrophils is higher than the average at day-14, indicating there is still higher activity to bacteria phagocytosis. The antimicrobial capacity of neutrophils is higher than that of macrophages [12, 23]. Therefore, these conditions give more effect to collagen synthesis only on the last healing process. The results showed that the infection is reduced in the PA group and does not disturb the proliferation phase significantly. Thus, the wound closure process using *S. duplicatum* improves the mechanism of angiogenesis and re-epithelization [24]. In addition, *S. duplicatum* has strong absorption and absorbs a lot of rapid exudates in a form of a gel that creates a moist environment and maintains the homeostatic condition of a wound to stimulate wound healing faster [12, 25]. The numbers of neutrophils and macrophages are not significantly different from normal control. It showed that the combination of *S. duplicatum* and *G. mangostana* has potential antimicrobial, antioxidant, and anti-inflammatory properties [26]. The antioxidants in *G. mangostana* reduce ROS and decrease the inhibition for migration and proliferation of fibroblasts, endothelial cells, and keratinocytes, and repair skin tissue better than diabetic conditions [12]. The results displayed that xanthone compounds can reduce cell toxicity and damage by preventing NF-*κ*B pathways and MAPK activation so that the re-epithelization proceeds smoothly [27]. Fibroblasts are the primary cells in the tissue reconstruction process in the proliferation phase due to collagen production needed for wound healing. There is a high possibility that the diabetic open wounds treated in combination with *S. duplicatum* and *G. mangostana* will have entirely healed before day 14 after the injury. Therefore, it is recommended to observe in 10–12 days after the injury for further research.

## 5. Conclusions

The topical administration of alginate extracted from *Sargassum duplicatum* and *Garcinia mangostana* combination can lead to re-epithelialization in the open wound of diabetic mice. Therefore, it can be concluded that the topical administration of alginate extracted from *Sargassum duplicatum* and *Garcinia mangostana* combination improved open wound healing in diabetic mice due to the antioxidants from xanthone and the polysaccharide polymer in alginate, which plays the role as an absorbent. Although the combination showed an improvement in open wound therapy, further study needs advanced methods, comparison with commercial drugs, and advanced variables such as NF-*κ*B and MAPK pathways to confirm the effectiveness of the *Sargassum duplicatum* and *Garcinia mangostana* combination.

## Figures and Tables

**Table 1:** Wound healing parameters on day 3, 7, and 14 of treatment.

Groups			Wound width (*μ*m)	Neutrophils (cells/nm^2^)	Macrophage (cells/nm^2^)	Fibroblast (cells/nm^2^)	Fibrocyte (cells/nm^2^)	Collagen density (%)
KN	Day	3	4849 ± 106.07	117.5 ± 5.42	12.5 ± 1.18	46.17 ± 7.78	21.33 ± 1.41	63.5 ± 4.01
	Day	7	1166.17 ± 20.03	88.17 ± 1.18	8.83 ± 0.24	73.67 ± 3.3	28.5 ± 1.65	80.83 ± 1.18
	Day	14	206.83 ± 7.78	31.5 ± 0.71	4.67 ± 0.47	45.83 ± 0.24	16.83 ± 1.18	93 ± 1.41

KD	Day	3	2642.17 ± 33.71^*∗*^	159.83 ± 6.36^*∗*^	17 ± 0.94^*∗*^	41.67 ± 10.84	13.17 ± 0.71^*∗*^	56.67 ± 1.89^*∗*^
	Day	7	6478.5 ± 320.32^*∗*^	132.67 ± 7.07^*∗*^	15.83 ± 4.01^*∗*^	51.83 ± 9.19^*∗*^	19.5 ± 0.71^*∗*^	60.83 ± 4.01^*∗*^
	Day	14	243.17 ± 10.61	57.83 ± 6.84^*∗*^	2.67 ± 0.47	38.83 ± 4.95	14 ± 0.94^*∗*^	89.67 ± 1.41

PA	Day	3	3374.5 ± 381.6^*∗*^	115.5 ± 7.31^+^	11 ± 0.94^+^	35.33 ± 1.89	17 ± 0.94^*∗*+^	66.67 ± 0.94^+^
	Day	7	1565 ± 447.83^+^	98.5 ± 8.72^+^	9.17 ± 0.71^+^	60.17 ± 3.54^*∗*^	26.67 ± 0.47^+^	87.5 ± 1.18^*∗*+^
	Day	14	162.33 ± 17.91	61.17 ± 24.75^*∗*^	2.17 ± 0.24	41.83 ± 1.18	14.5 ± 0.24^*∗*^	90.5 ± 0.71

PAM	Day	3	4701.67 ± 68.35^+^	131.67 ± 9.43^+^	11.83 ± 0.71^+^	35.17 ± 4.01	21.83 ± 0.71^+^	68.83 ± 0,71^*∗*+^
	Day	7	1512.83 ± 27.11^+^	114.83 ± 9.66^*∗*^	8.67 ± 0.47^+^	67.83 ± 0.71^*∗*+^	30.5 ± 1.18^+^	82.17 ± 2.59^*∗*+^
	Day	14	136.5 ± 4.01	48.17 ± 0.24	1.83 ± 0.24	56 ± 7.07^+^	17.33 ± 1.41^+^	93.83 ± 2.12

Note: The data are expressed as the mean ± SD (*n* = 6). ^*∗*^*p* < 0.05 compared to the normal control group. ^+^*p* < 0.05 compared to the diabetic control group. KN: normal control, KD: diabetic control, PA: *S. duplicatum* extract treatment, and PAM: combination of *S. duplicatum* and *G. mangostana* extracts treatments.

## Data Availability

The data used to support this study are available from the corresponding author on reasonable request.
